# Code-switching functions in online advertisements on Snapchat

**DOI:** 10.1371/journal.pone.0287478

**Published:** 2023-07-20

**Authors:** Mohammad Almoaily

**Affiliations:** Department of English Language, College of Language Sciences, King Saud University, Riyadh, Kingdom of Saudi Arabia; Tallinn University: Tallinna Ulikool, ESTONIA

## Abstract

Code-switching is a well-researched phenomenon. However, little research has been conducted on code-switching in orally communicated advertisements on social media despite social media platforms witnessing the birth of many linguistic trends and having an impact on the linguistic behaviour of their users. To bridge this gap, the current study investigates the functions of Arabic-English code-switching in advertisements made by Saudi influencers on Snapchat. The data comprise 4000 words produced by 40 advertisers (20 male and 20 female, 100 words each). The advertisers, who belong to the same age group, advertised for a range of products and targeted mostly monolingual Saudi users of Snapchat. The sample produced 102 instances of switching from Arabic to English. The most common function in the data consisted of switching for availability (n = 78), but other functions were observed (quotation n = 9, specifying addressee n = 5, interjection n = 7, reiteration n = 1, and message qualification n = 2). The potential reasons for the higher frequency of switching for availability are discussed, and venues for future research are suggested.

## Introduction

Code switching, i.e., using more than one language in one setting [1], is a widely attested feature in the speech of bilingual people. Given its high frequency in various domains of language use, such as education, trade, and family, it is not surprising that it has gained the interest of researchers from various subdisciplines of linguistics. Indeed, [2] report that this phenomenon has been studied from different perspectives, such as syntax (e.g., [3]’s three types of switching—intersentential, intrasentential, and tag switches—in addition to other works on syntactic constraints on code switching, such as [4–6], sociolinguistics ([7, 8]), and psycholinguistics ([9, 10]). One of the areas of study in the topic of code-switching consists of the reasons for which people switch between languages and more particularly the social functions for which speakers switch codes. Alternating between two languages in a single discourse can serve various functions, which can be classified under two main categories: *situational* and *metaphorical*. In the first category, switching between two or more languages results from a change in the social situation, such as the arrival of a new person or a change in the degree of discourse formality. Metaphorical code-switching, in contrast, occurs when speakers switch codes to enrich the situation, such as expressing emotions or showing expertise in a topic (see [11, 12]). These functions are detailed in the literature review section.

Although the functions of code-switching for social reasons have been widely studied, very few sociolinguists have addressed the functions of code-switching on social media platforms, particularly Snapchat. Therefore, the current study focuses on the sociolinguistic functions of code-switching in orally communicated advertisements on Snapchat. This area of research is worthy of further investigation for two main reasons. First, very few studies have addressed the functions of code-switching on this social media platform despite its influence on social media users and the large community of users (over 360 million active users in the third quarter of 2022, according to [13]). Second, the communication between speakers and their audience is asynchronous, making it different from the well-studied functions of code-switching in synchronous communication. Hence, the current study addresses the following question:

1. What are the functions of code-switching in orally communicated ads on Snapchat?

The following section discusses the related literature. This is followed by the methods section, in which the sample, data, and procedures are detailed. The data are then displayed in the results section. Finally, the discussion section delves into the findings of the current study. The limitations and recommendations for future research are discussed in the conclusion.

## Literature review

Code-switching has long been stigmatised as an impure form of speech and is still defamed by some speech communities. [12] reported some of these negative labels of code-switching (e.g. Tex Mex, joual, and tuti fruti) as a proof for the negative attitudes towards code-switching by some speech communities. This negative labelling of code-switching, according to [14: 170], was shaped by the “notions of linguistic purism that have dominated Western thought about language for hundreds of years”. It was not until the 1960s that linguists, led by the works of John Gumperz, started to regard this phenomenon as a skilful form of speech involving a number of social functions. The sections below address these functions and the controversies surrounding them. Additionally, a review of studies of code-switching in advertisements and Arabic-English code-switching is provided. Finally, the contribution of the current study is discussed in light of the gap in the literature that the study seeks to bridge.

### Sociolinguistic and social functions of code-switching

Two of the earliest attempts to link code-switching with social functions were [11, 15], in which this linguistic phenomenon was categorised into situational and metaphorical switching. The former covers an array of instances of switching between two languages due to a change in the situation, including the arrival of a new person in a given dialogue, the starting of a foreign language class, and a change in the formality of the discourse (e.g., from casual to religious domain). Metaphorical switching, in contrast, is not predicted by a change in the situation but rather is done to achieve social functions, such as assigning group membership or switching to express feelings and emotions.

Based on these two types of code-switching, [15] listed six functions of code-switching. The first function is switching to another language to quote others to ensure that the quotation is reported accurately because translation can alter meaning in one way or another. The second function specifies addressees, i.e., code-switching when speaking to different addressees. The third function is interjection, which is achieved via the insertion of expressions or phrases from another language as sentence fillers. The fourth function is reiteration, which serves the function of repeating one’s own words in a different language to clarify or emphasise one’s message. The fifth function is message qualification, and the sixth is personalisation vs. objectification (also referred to as the we vs. they codes). As suggested by this labelling, the switching between languages serves the function of either talking about oneself (code 1) or talking about others (code 2). [2] also suggested a list of code-switching functions. The first is referential code-switching, in which the speaker switches to another language as a speech remedy technique when he or she fails to continue speaking in one language. This function can occur due to incompetency, lack of knowledge, or any other reason resulting from lack of knowledge about the subject being discussed. In the current study, this type of switching is referred to as switching for availability. The second function is directive, which is quite similar to [15]’s aforementioned function *addressee specification*. The third function suggested by [2] is switching for expressive functions, that is, switching to show one’s ability to speak in two languages. The fourth function is phatic switching, in which speakers switch to change the tone of the conversation, mostly to place more emphasis on important information by restating it in another language. The fifth function is metalinguistic, as when a speaker comments on a statement that he or she produced in L1 using L2. The last function is poetic function, in which a bilingual speaker switches between languages for aesthetic functions, such as telling jokes and making puns.

Although the situational versus metaphorical dichotomy in the classification of the types of code-switching has changed the way in which many linguists perceive using more than one language in a single discourse, this binary distinction has received some criticism. For instance, [16] argued that the distinction between these two types of code-switching is unclear, making it difficult to classify certain instances of switching as either situational or metaphorical. This criticism for Gumperz’s situational vs. metaphorical switching is also found in [17–19: 120] even opposed the practice of listing functions of code-switching, suggesting that the “mere listing of such loci is problematic”. He justified this claim by listing a number of reasons why devising a finite list of code-switching functions is problematic. First, these functions and categories are not properly defined and thus are confusing to researchers. As an example, he suggested that a function such as reiteration fails to account for the many potential meanings of this linguistic behaviour. He also suggested that these typologies fail to distinguish among conversational structures, linguistic forms, and functions of code-switching (code-alternation in [19]’s terms). [19] also questioned whether listing the code-switching functions leads to a solid theory, more specifically whether the lists of functions would convey the meaning of this linguistic behaviour. Finally, he criticised these lists of providing the same social and linguistic value for switching from language A to B or vice versa. As an alternative, [19] proposed an alternative model, suggesting that code-alternation should be conceived of as a contextualisation cue, similar to prosodic and gestural cues in monolingual conversations. Hence, [19] suggested that participants alternate between languages to “make relevant/maintain/revise/cancel some aspects of context which, in turn is responsible for the interpretation of an utterance in its particular locus of occurrence” ([19: 123]).

Although the aforementioned criticisms of listing the functions of code-switching suggest that there can be infinite functions of code-switching; they cannot escape listing aspects of contextualisation that, in turn, serve as code-switching functions. That proposing lists of code-switching functions seems inevitable can be a strong argument in favour of [2, 11] lists of code-switching functions, not only because they provided a list of the most common types of code-switching but also for the seemingly unavoidable necessity for listing the functions, even among those who object to this practice. [14] also argued that [19]’s two categories, discourse-related switching and participant-related switching, rearticulate [15] conversational and situational code-switching. It is not surprising, then, that [20] argued that [15] framework is still a dominant and well-cited framework in the literature on code-switching. Therefore, the current study adopts a middle position by taking [11]’s distinction and functions as a theoretical framework while bearing in mind that online verbal communication might exhibit slightly different linguistic behaviours, calling for scrutinising and revising the well-established literature on code-switching in conventional (face-to-face) communication.

### Code-switching in social media

Code-switching is not only found in physical face-to-face interactions. It can indeed be found in online interactions via social media platforms. According to [21], social media is a broad term covering a range of websites and applications that enable internet users to share content in various forms, such as text, images, audio, and video. Content on social media is a great candidate for naturalistic studies such as the current one for a number of reasons. First, the linguistic data on social media are recent and thus reflect the current linguistic practices of the targeted speech community, which is especially important since social media influencers are followed by a large population of youths and can indeed be important actors in the process of language change. Second, the content created by individuals is more likely to be natural than that created by professional advertisers because professionals have more time and experience to create linguistically revised and rehearsed advertisements. Individuals, conversely, are less careful about revising their linguistic content, especially in applications that erase content after a short period, such as Snapchat. It is not surprising, then, that some individuals have been found to code-switch more frequently in social media than in traditional media (Karlsen’s [unpublished]). Additionally, see [22] for more discussion of the value of social media content in linguistic studies using naturalistic data.

Many studies have approached the topic of code-switching using social media content. For instance, [23] analysed English-Indonesian code-switching types (i.e., intrasentential, intersentential, and tag switching) in podcasts. The study found that intrasentential code-switching was the most frequent of the three. Alrashed (unpublished) inspected the frequency and functions of Arabic-English code-switching in podcasts discussing technology. Although the code-switching instances were very low in her data, Alrashed reported that podcasters code-switched for various functions, such as repair, contrast, and narration. [24] reported that Indonesian Instagram users code-switch between English and Indonesian as a way of shaping a well-educated identity. [25] also reported that educated Egyptian Arabic speakers code-switch between Arabic and English to save time and to express emotions. [26] study found that code-switching was used by Arabic speakers to serve an array of functions, such as clarification, emphasis, and expressing emotions. Additionally, see [27, 28], and Ria (unpublished) for analyses of code-switching in social media. However, as far as the researcher is aware, there have been no previous attempts to consider the functions of code-switching functions on Snapchat. Therefore, the current study is an attempt to bridge this gap by analysing the functions of Arabic-English code-switching in advertisements on Snapchat made by Arabic speakers.

### Code-switching in advertising

Advertisers use various techniques to convince consumers to buy the products for which they advertise. Most of these techniques are nonlinguistic (see [29–31]). However, the linguistic aspects of advertisements can also be important. For instance, the language or language variety used in advertisements might have an impact on consumers’ perception of the advertisements. In addition, using a catch phrase or switching between two languages can be a persuasive tool for advertisers. Hence, code-switching is reported to be common in advertisements, especially in bilingual communities. For instance, [32] examined 50 advertisements and found that code-switching between English and Sinhala was used in nearly 50% of them. In the same vein, [33] analysed a sample of 135 advertisements in Bengali and found that only 13 of them were Bengali monolingual, while the rest contained English code-switching. Switching between varieties of Arabic in written commercials, in addition to Arabic-English code-switching, was also reported by [34].

Switching between languages in advertisements can have various functions that, in turn, help advertisers to convince the targeted audience. For instance, [35] reported that switching from a minority language to a majority language leads to positive evaluations for the advertised product. Additionally, [36] reported that code-switching between German and English attracted negative evaluations of advertisements. However, the majority of her sample still viewed commercials containing code-switching as more modern than German monolingual ads. [37] also reported that English is used in advertising in Russia as a manifestation of modernity and prestige. Moreover, [38] reported that switching between English and Macedonian was conceived of as a tool to improve the lexical and syntactic properties of 200 sampled commercials. Furthermore, [39] suggested that advertisers in the Philippines utilised code-switching for the purposes of advising, describing, explaining, and illustrating; language economy; euphemism; and the portrayal of multiple identities.

Most of the studies reviewed above investigated advertising in bilingual or multilingual communities. It should be noted, however, that code-switching is not common in advertisements targeting mostly monolingual speakers, as reported in the current study; see also [40]. Additionally, investigations of code-switching in advertisements on social media have been scarce, resulting in calls for more research on online advertisements, especially those made spontaneously by social media influencers. Hence, the current study is an attempt to bridge this gap by targeting code-switching functions in Snapchat, which are directed towards mostly monolingual Saudi consumers.

## Methods

**Fig 1:**
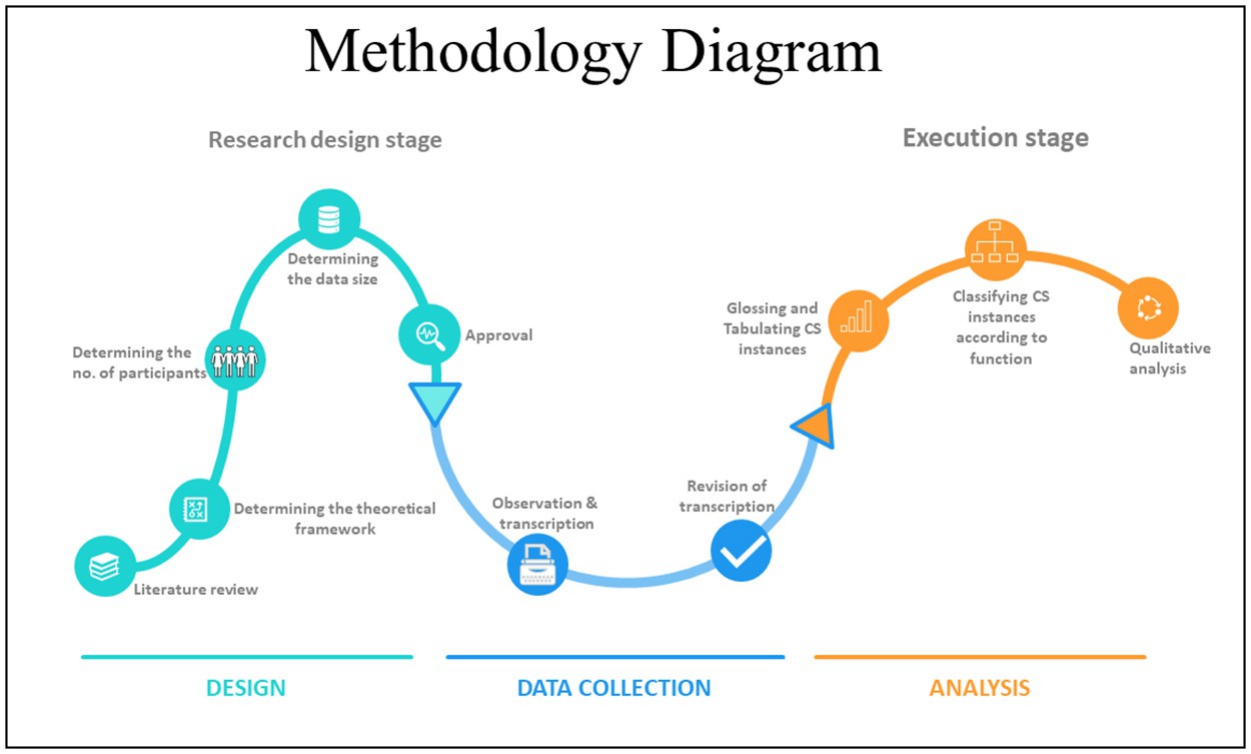
Methodology diagram.

The study aimed to determine the functions of code-switching in advertisements made by Saudi influencers on Snapchat. To achieve this aim, a number of procedures were implemented. First, a survey of the literature was made to determine an appropriate theoretical framework for the functions of code-switching in social media. Based on the discussion of the sociolinguistic and social functions of code-switching in the literature review section above, [2, 11] theory of code-switching as a social practice was followed. The lists of code-switching functions set forth by this theory were used in the current study as references for classifying the instances of code-switching in the data into their relevant functions (the functions *quotation*, *specifying addressee*, *interjection*, *reiteration*, *message qualification*, and *availability* are defined in the literature review section) Second, a corpus was compiled using a sample of advertisements (see the sample section below). The data, detailed in a section below, were transcribed and instances of code-switching were highlighted and were then classified according to functions. Finally, the data were analysed. Fig 1 provides an illustration of the methodology implemented in the current study, from the first step in the research design stage (i.e., choosing the sociolinguistic theory as a theoretical framework), to the last step in the execution stage (i.e. discussing the data qualitatively). All the advertisements included in the analysis were communicated verbally and were spontaneous in nature. The study design was approved by the appropriate ethics review board at King Saud University. Since the data were publicly available, the need for consent was waived. The sub-sections below discuss the sample, data, instruments, and procedures of the study in detail.

### Sample

Given the relatively limited number of Saudi influencers on Snapchat who regularly advertise on the platform and to collect balanced data from both male and female influencers, cluster sampling was employed to collect data from 40 Saudi Snapchat advertisers (20 male and 20 female). To increase the accuracy of the data, the sampled participants had to meet certain criteria to be included in the study. First, their targeted audience was the same (Saudi locals, i.e., the influencer’s Saudi Snapchat followers). Hence, advertisements targeting non-Arabic speaking expatriates in Saudi Arabia or made by Saudis living abroad were not included in the analysis since the targeted audience is different, which could lead to discrepancies in the data. Additionally, the advertisers were from the same age range (young adults). In addition, all of the sampled advertisers had to be regularly advertising since doing so increased the spontaneity of the data. Snapchat influencers who are not accustomed to advertising on a regular basis might change their linguistic behaviour.

### Data

The advertisers included in the study advertised for various products and services, including but not limited to beauty and fashion, home appliances, cars, clothing, and food and drinks. All advertisements included in the study were made in the months of September and October (2022). To collect an even amount of data from each participant, the first one hundred words in each advertisement made by the 40 advertisers were transcribed (see the Procedures section below) and included in a corpus comprising 4000 words. Hence, each sampled advertiser contributed to this corpus with one hundred words, also meaning that half of the corpus was produced by men and the other half was produced by women. Including only the first one hundred words in each advertisement was an additional measure taken to increase the homogeneity of the data because speakers’ linguistic behaviours can differ at different stages of a dialogue (i.e., initial vs. final).

### Procedures

As mentioned in the previous section, the advertisements were transcribed to facilitate the analysis of the results of the current study. Since the transcription had to be accurate, it was undertaken in two stages. First, Microsoft Word’s dictation command was used to create a first draft of the transcription of each advertisement. Since the first step created a nearly accurate version of the transcription, there was a need to watch the advertisements again and to edit the transcriptions manually to reach the highest level of accuracy. Instances of code-switching were highlighted to facilitate the process of analysing their functions based on the context in which they appeared. It should also be noted that lexical borrowing instances were not regarded as instances of code-switching since such words have been added to the Arabic lexicon and are no longer considered foreign words. Additionally, brand names in English that do not have Arabic translations by the trademark owners were not considered code-switching cases. Including a specific number of words from each participant, rather than minutes of speech, has the benefit of collecting an even amount of data from each individual in the sample, which is especially important in the current study because advertisers might have long pauses in their speech while unpacking products or showing how they are used.

Another important procedure implemented in the current study is ensuring the safety of the sample and protecting the participants’ personal information. Although all of the sampled advertisers make public advertisements for safe use of products and services, their real names and identities are not reported in the current study. Instead, labels are used when providing examples of the functions observed in the data (e.g., F1 for the first female advertiser included in the corpus and M3 for the third male advertiser).

## Results

The functions of code-switching in the data are displayed in Table 1 below.

**Table 1:** Code-switching functions in Snapchat ads.

Function	No. of tokens	Percentage
Quotation	9	8.8%
Specifying addressee	5	4.9%
Interjection	7	6.8%
Reiteration	1	0.9%
Message qualification	2	1.9%
We/they	0	0%
Availability	78	76.5%
**Total**	102	

The total number of code-switching instances in the 4000-word corpus was only 102. This limited number of code-switching instances is expected in a mostly monolingual speech community. Switching for availability was the most frequent type of switching in the data (76.5%). This type of code-switching refers to one-word switches done to avoid the difficult task of looking for accurate equivalents in the language that the speaker is speaking ([41]). Other code-switching functions were even less frequent (e.g., quotation, 8.8%, specifying addressee, 4.9%, and interjection 6.8%). Some of the functions of we/they were not observed in the data.

These findings are further discussed below, but first, we look at some examples from the data. Please note that the first line in each example is a transliteration of the example, which was originally transcribed in the corpus in Arabic, while the code switch to English is in bold. The second line provides glosses of the first line, and the third line is a translation of the example:

1. *Quotation*
  Example no. 1 (Speaker: F17)

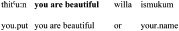

  You put “you are beautiful” or your name.
  Example no. 2 (Speaker: F20)

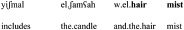

  It (the offer) includes the candle and the hair mist.
2. *Specifying Addressee*
  Example no. 1 *(Speaker*: *M14)*

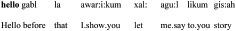

  Hello, before I show you let me tell you a story.
  Example no. 2 *(Speaker*: *F4)*

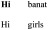

  Hi girls
3. *Interjection*
  Example no. 1 (Speaker: M19)

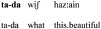

  ta-da! What’s this beautiful *thing*?!
  Example no. 2 (Speaker: M12)

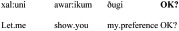

  Let me show you my preference, OK?
4. *Reiteration (Only one instance was found in the data produced by F10)*

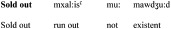

  Sold out, run out, inexistent!
5. *Message Qualification (Speaker M7)*

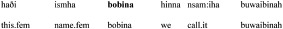

  This is a bobina ‘ignition coil’, but we call it ‘buwaibinah’.
6. *Availability (Speaker F3)*
  Example no. 1 (Speaker: F3)

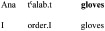

  I have ordered gloves.
  Example no. 2 (Speaker: M9)

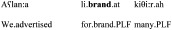

  We advertised for many brands.

The section below discusses these findings in more detail.

## Discussion

The results displayed in the previous section showed that Saudi Snapchat advertisers mainly use Arabic in their advertisements, but they occasionally switch to English. The instances of code-switching, albeit infrequent, served all the functions set forth by [15], with the exception of *we/they*, which was not used by any of the 40 advertisers polled in the study. Another interesting finding is that the tokens were not evenly distributed across the six functions observed in the data. The function of *availability* was used much more than all the other functions combined, at 76.5%, while some functions were scarcely employed (e.g., reiteration, 0.9% and message qualification 1.9%).

The limited number of instances of code-switching (102) in a 4000-word corpus can be explained by the advertisers being mostly Arabic monolinguals, and the audience whom they target are also mostly Arabic monolingual. The limited occurrence of code-switching instances by monolingual social media influencers was also reported in [23] study, in which an Indonesian podcaster switched to English only nine times. The researchers, however, did not specify their corpus size. Additionally, Alrashed (unpublished) reported a limited number of Arabic-English code-switches by Saudi podcasters, with the percentage of English words ranging between 4% and 15%, although the topics discussed in the podcasts were relevant to technology—a topic that was expected to have a high rate of switching to English.

The higher rate of switching for availability can be explained by a number of reasons. First, advertisers might use English words if they provide a shorter alternative than Arabic words. For instance, the English word *brand* was used 17 times, while its longer Arabic equivalent *ʕala*:*mah tidʒarijah* only occurred twice in the data. It is not surprising that speakers prefer the shorter, easier-to-pronounce alternative over the longer version. Second, advertisers might deliberately switch to English to make a product more appealing to viewers ([35]) or to have it appear more modern ([36]). Additionally, other functions, such as we vs. they, are rarely needed when the speaker and the addressee belong to the same ethnicity and have the same linguistic background. Specifying addressees is also hardly needed on Snapchat, where the speaker speaks to a virtual audience. Additionally, other functions, such as *reiteration* and *message qualification*, require a level of English proficiency that would enable the speaker to explain what he or she said in a different language. Those with limited English proficiency are indeed not expected to reiterate their speech in English, which would take more time and would render the advertisement boring to viewers. Message qualification is also not expected to occur frequently since there is no real need to explain one’s speech in a different language if all the expected audience would fully understand it without resorting to English for clarification or more elaboration.

The sampled men and women did not show different behaviours in terms of the functions of code-switching. For instance, both groups code-switched for availability more frequently than for other functions (73.8% by men and 78.3% by women). Similarly, we/they were not used by any member of the two groups. Message qualification was used twice by one male participant. None of the remaining male advertisers or the female advertisers code switched to English for message qualification. All instances of code-switching for quotation were produced by female speakers. One potential reason for more code-switched quotations by the women is the advertised product, with accurate descriptions of the colour of the contact lenses being important to the audience, as in the data produced by F9 and the skin care products by F20.

### Implications

One of the major findings of the current study is that switching for availability is the most common type of code-switching in the data (76.5%). This is despite that most of sampled advertisers are either Arabic monolinguals or speak English with limited proficiency. It indeed seems counterintuitive, as switching for availability generally requires a great deal of competency in both the matrix and the embedded languages. Yet, the dominance of switching for availability in the corpus can be explained by Arabic corpus planners’ slow pace in suggesting appropriate translations for emerging concepts. Hence, Arabic speakers are going to be exposed to the English term well before the Arabic alternative is made available. Hence, one of the implications of the current study is to shed light on this problem and to call for increased efforts in Arabic translation by both organisations and individuals.

Another implication is peculiar to classifying code-switching functions. Coming up with lists of functions has, as detailed in the literature review section, been a matter of controversy as coming up with a limited list of functions seems impossible. Arguments against limited lists of functions are supported by the findings of the current study. This is due to the fact that the most frequent type found in the data (i.e., switching for availability) was not even included in [15], which is one of the most cited lists of code-switching functions in the literature.

## Conclusion

This study aimed to determine the functions of code-switching in advertisements communicated verbally over the social media platform Snapchat. To achieve this aim, a corpus containing 4000 words produced by 40 female and male advertisers (100 words per advertiser) was used to calculate and classify the instances of code-switching into a range of social and sociolinguistic functions. Switching for availability was the most common function for code-switching in the data. Other functions were rarely employed or even not used by any of the sampled advertisers.

Although careful measures were undertaken to ensure polling data from a homogenous sample (e.g., all the sampled advertisers belonged to the same age group, are Saudis who reside in Saudi Arabia and advertise to Arabic-speaking audience), some variables were difficult to control. For example, determining the exact English level of each advertiser was not possible since doing so would require requesting that each provide an English proficiency certificate. Additionally, unifying of the products that advertisers promote is difficult to achieve, given the limited number of advertisers.

Further investigation of the topic could include attitudes towards code-switching in social media and the impact of the mode of delivery (verbal vs. written) on the frequency of certain code-switching functions. Another promising area of research is the potential discrepancy of the frequency of code-switching and its functions by different age groups.
